# A microfabricated, 3D-sharpened silicon shuttle for insertion of flexible electrode arrays through dura mater into brain

**DOI:** 10.1088/1741-2552/ab2b2e

**Published:** 2019-10-29

**Authors:** Hannah R Joo, Jiang Lan Fan, Supin Chen, Jeanine A Pebbles, Hexin Liang, Jason E Chung, Allison M Yorita, Angela C Tooker, Vanessa M Tolosa, Charlotte Geaghan-Breiner, Demetris K Roumis, Daniel F Liu, Razi Haque, Loren M Frank

**Affiliations:** 1 Medical Scientist Training Program and Neuroscience Graduate Program, University of California, San Francisco, CA 94158, United States of America; 2 Kavli Institute for Fundamental Neuroscience, Center for Integrative Neuroscience, and Department of Physiology, University of California, San Francisco, CA 94158, United States of America; 3 Bioengineering Graduate Program, University of California Berkeley/University of California, San Francisco, CA 94158, United States of America; 4 Lawrence Livermore National Laboratory, Center for Micro- and Nano-Technology, Livermore, CA 94550, United States of America

**Keywords:** polymer electrode arrays, silicon electrode arrays, durotomy, chronic neural recording, rat, multi-electrode arrays

## Abstract

**Objective.:**

Electrode arrays for chronic implantation in the brain are a critical technology in both neuroscience and medicine. Recently, flexible, thin-film polymer electrode arrays have shown promise in facilitating stable, single-unit recordings spanning months in rats. While array flexibility enhances integration with neural tissue, it also requires removal of the dura mater, the tough membrane surrounding the brain, and temporary bracing to penetrate the brain parenchyma. Durotomy increases brain swelling, vascular damage, and surgical time. Insertion using a bracing shuttle results in additional vascular damage and brain compression, which increase with device diameter; while a higher-diameter shuttle will have a higher critical load and more likely penetrate dura, it will damage more brain parenchyma and vasculature. One way to penetrate the intact dura and limit tissue compression without increasing shuttle diameter is to reduce the force required for insertion by sharpening the shuttle tip.

**Approach.:**

We describe a novel design and fabrication process to create silicon insertion shuttles that are sharp in three dimensions and can penetrate rat dura, for faster, easier, and less damaging implantation of polymer arrays. Sharpened profiles are obtained by reflowing patterned photoresist, then transferring its sloped profile to silicon with dry etches.

**Main results.:**

We demonstrate that sharpened shuttles can reliably implant polymer probes through dura to yield high quality single unit and local field potential recordings for at least 95 days. On insertion directly through dura, tissue compression is minimal.

**Significance.:**

This is the first demonstration of a rat dural-penetrating array for chronic recording. This device obviates the need for a durotomy, reducing surgical time and risk of damage to the blood-brain barrier. This is an improvement to state-of-the-art flexible polymer electrode arrays that facilitates their implantation, particularly in multi-site recording experiments. This sharpening process can also be integrated into silicon electrode array fabrication.

## Introduction

Electrode arrays for implantation in the brain are a technology critical to both fundamental neuroscience and clinical treatments for diseases including epilepsy (Toth *et al* 2016), retinal degeneration (Luo and da Cruz 2016), Parkinson’s, and depression (Lozano *et al* 2019). Classically, electrode arrays have been made of silicon (Rousche and Normann 1998, Kipke 2003) or other hard metal (Nicolelis *et al* 2003). While these devices can be effective in recording single units (Mols *et al* 2017), the longevity of recordings is limited (Polikov *et al* 2005, Jeong *et al* 2015). More recent designs of silicon electrodes with smaller cross sections can record an estimated one cell per electrode, and can detect single units in mouse for at least 150 d (Jun *et al* 2017), but the ability to record continuously from a given neuron over days has yet to be demonstrated. These limitations are thought to result from the stiffness of the device relative to brain tissue.

This has inspired the development of flexible neural implants. These include probes (Sohal *et al* 2014, Jeong *et al* 2015, Xie *et al* 2015, Fu *et al* 2016, Chung *et al* 2019) and mesh (Zhou *et al* 2017) that are better matched mechanically to brain tissue and show reduced inflammation and immune responses (Szarowski *et al* 2003, Harris *et al* 2011, Jeong *et al* 2015, Lacour *et al* 2016, Lee *et al* 2017, Hong and Lieber 2019), particularly if they are also small (Seymour and Kipke 2007, Kozai *et al* 2012, Luan *et al* 2017). Chronically implanted in animal models, polymer devices can yield single-cell recordings spanning months (Jeong *et al* 2015, Fu *et al* 2016, Luan *et al* 2017, Chung *et al* 2019), and many of the same individual neurons can be recorded continuously for at least ten days (Chung *et al* 2019).

As penetrating neural devices are made smaller and more compliant to reduce tissue damage, surgical technique becomes more complicated. The force required to insert a flexible device into the brain typically exceeds the critical buckling load of the device. This presents a major barrier to use for many flexible devices (Lecomte *et al* (2018) and Weller *et al* (2018) are cited in text but not provided in the list. Please provide complete publication details to insert in the list, else delete the citation from the text.]). Insertion is made more difficult as a result of the dense irregular connective tissue—the dura, arachnoid, and pia mater—that together protect the brain from mechanical and other insults (Weller *et al* 2018).

Solutions to the problem of inserting a flexible electrode array into the brain involve surgical removal of the dura mater, then insertion through the remaining pia by methods that temporarily increase the critical load of the device using biodissolvable coatings (Kozai *et al* 2014, Kim *et al* 2013, Xiang *et al* 2014, Lo *et al* 2015, Patel *et al* 2015, Wu *et al* 2015, Khilwani *et al* 2016), fluidic injection (Takeuchi *et al* 2005, Liu *et al* 2015, Schuhmann *et al* 2018, Vitale *et al* 2018), freezing (Xie *et al* 2015), or shape memory polymer (SMP; Simon *et al* 2017). One straightforward solution is to temporarily attach the flexible array to a hard shuttle, such as silicon (Kozai and Kipke 2009, Felix *et al* 2012, Patel *et al* 2015, Chen *et al* 2017), or tungsten (Zhao *et al* 2019) that is shape-matched to the device itself and can be retracted once the array has been inserted to its target depth in the brain. Temporary attachment can be achieved by inserting shuttles through a small hole in the array (Hanson *et al* 2019) or, more commonly, by a biodissolvable adhesive. An advantage to this general method is that a very stiff material can be used to fabricate the insertion shuttle, since it will not be left in the brain. The shuttle diameter can thus be smaller, and the paired device very flexible, for reduced damage to the tissue on implantation and over the lifetime of a chronic implant (Seymour and Kipke 2007). Previously, we have used such a method to insert 16 independent polymer devices through rat pia, each to a different brain area, for high-quality, chronic recording (Chung *et al* 2019).

If a flexible device could be inserted directly through dura with minimal brain compression, then the tissue damage and longer surgical time that result from durotomy itself would be eliminated. Furthermore, a dural-penetrating method could also avoid errors in depth targeting that result from outward swelling of the brain following durotomy. Together, these effects could yield high quality, chronic single-unit recordings.

Of the previously mentioned insertion methods, only insertion using a delivery shuttle can be easily adapted for transdural delivery. For a shuttle made of a given material, with length determined by the depth of the target brain area, a lower limit on its cross-section is calculable by Euler’s critical load equation. A thicker device will have a higher critical load to more likely penetrate the meninges, but is not desirable because it will compress a greater area of brain tissue on insertion and disrupt more vasculature (Obaid *et al* 2018). One way to enable insertion of a device without increasing its diameter is to reduce the effective force on the device (i.e. to lower the required insertion force) by fabricating a sharper tip (Edell *et al* 1992, Bjornsson *et al* 2006, Jensen *et al* 2006, Sharp *et al* 2009).

Previous work has demonstrated the benefits of sharpened silicon device tips in reducing insertion force (Sharp *et al* 2009, Fekete *et al* 2015, Obaid *et al* 2018) and penetrating dura (Hosseini *et al* 2007, Fekete *et al* 2015). A diamond (Na *et al* 2018) delivery shuttle for polymer devices has demonstrated successful insertion of flexible polymer devices through rat dura, as has use of an insertion guide with an SMP device (Shoffstall *et al* 2018). However, such methods have not yet yielded chronic, single-unit recordings in freely behaving animals. We therefore aimed to develop a low-diameter, sharpened shuttle for polymer array delivery that could penetrate dura with minimal brain compression and yield high-quality single unit recordings.

Here, we describe a fabrication method which sharpens silicon shuttles in three dimensions at any tip angle or crystal orientation and which uses readily available cleanroom tools. We demonstrate a reduction in insertion force compared to planar-sharpened silicon shuttles on insertion through rat dura. Finally, we show that compliant devices implanted by transdural shuttle delivery yield high-quality recording of local field potential (LFP) and single units over months in the awake behaving rat. This is the first *in vivo* demonstration of successful chronic neural recording using a method to implant through a membrane with Young’s modulus in the range 0.1–1 MPa (Maikos *et al* 2008), corresponding to the rat dura or primate pia. 3D-sharpened shuttles obviate the need for a durotomy, increasing the efficiency and reliability of insertion for polymer electrode arrays to that of dural-penetrating arrays while maintaining the desirable properties of polymer for high quality single-unit neural recordings. Importantly, our fabrication method can be readily adapted to provide similar benefits to silicon-based multielectrode arrays.

## 1. Materials and methods

We fabricated a sharpened shuttle for insertion of flexible, thin-film polymer probes and tested transdural insertion *in vivo* in the rat. *In vivo* tests and chronic recording were performed in 10–16 month-old, male Long Evans rats. To evaluate this method, we measured insertion force and brain compression, which have been shown to predict neural tissue damage (Dixon *et al* 1991). First, we compared the insertion force through intact dura for shuttles that were either sharpened in three dimensions (sharpened) or two dimensions (planar) with otherwise identical dimensions. We first tested insertion through dura over the hippocampus, then tested insertion through the thicker dura over the orbitofrontal cortex (OFC). Second, we calculated the brain compression in each case. Finally, we implanted polymer devices using sharpened shuttles through intact dura over the nucleus accumbens (NAc) and OFC, and recorded LFP and single units for 95 d.

### 1.1. Shuttle microfabrication

**Figure 1.**
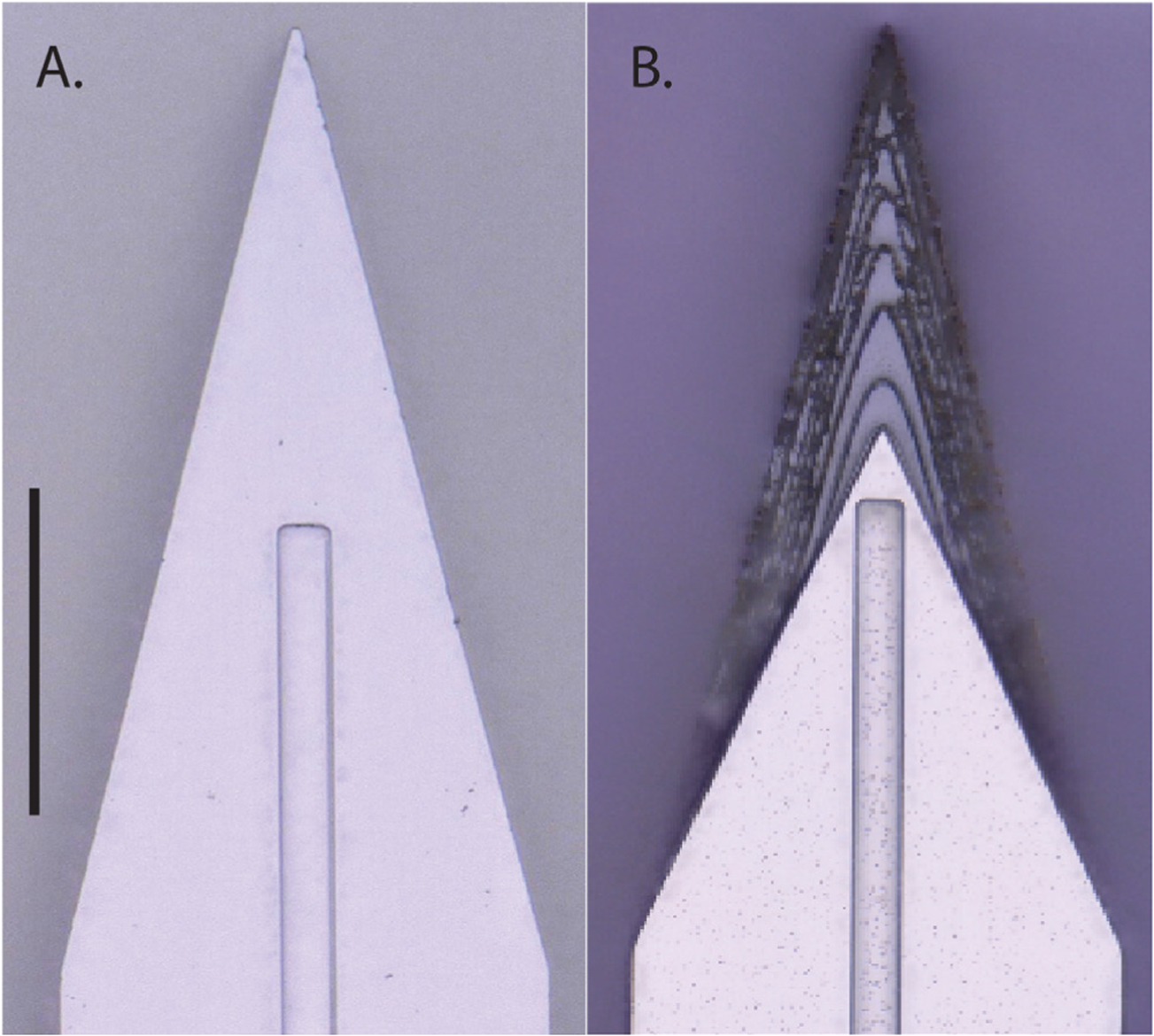
Top down view by light microscope (scale bar = 50 *μ*m) of (A) planar and (B) sharpened profiles on 2d- and 3d-sharpened silicon shuttles, respectively, oriented with tips toward the top of the figure, and wicking channel for PEG at midline.

**Figure 2.**
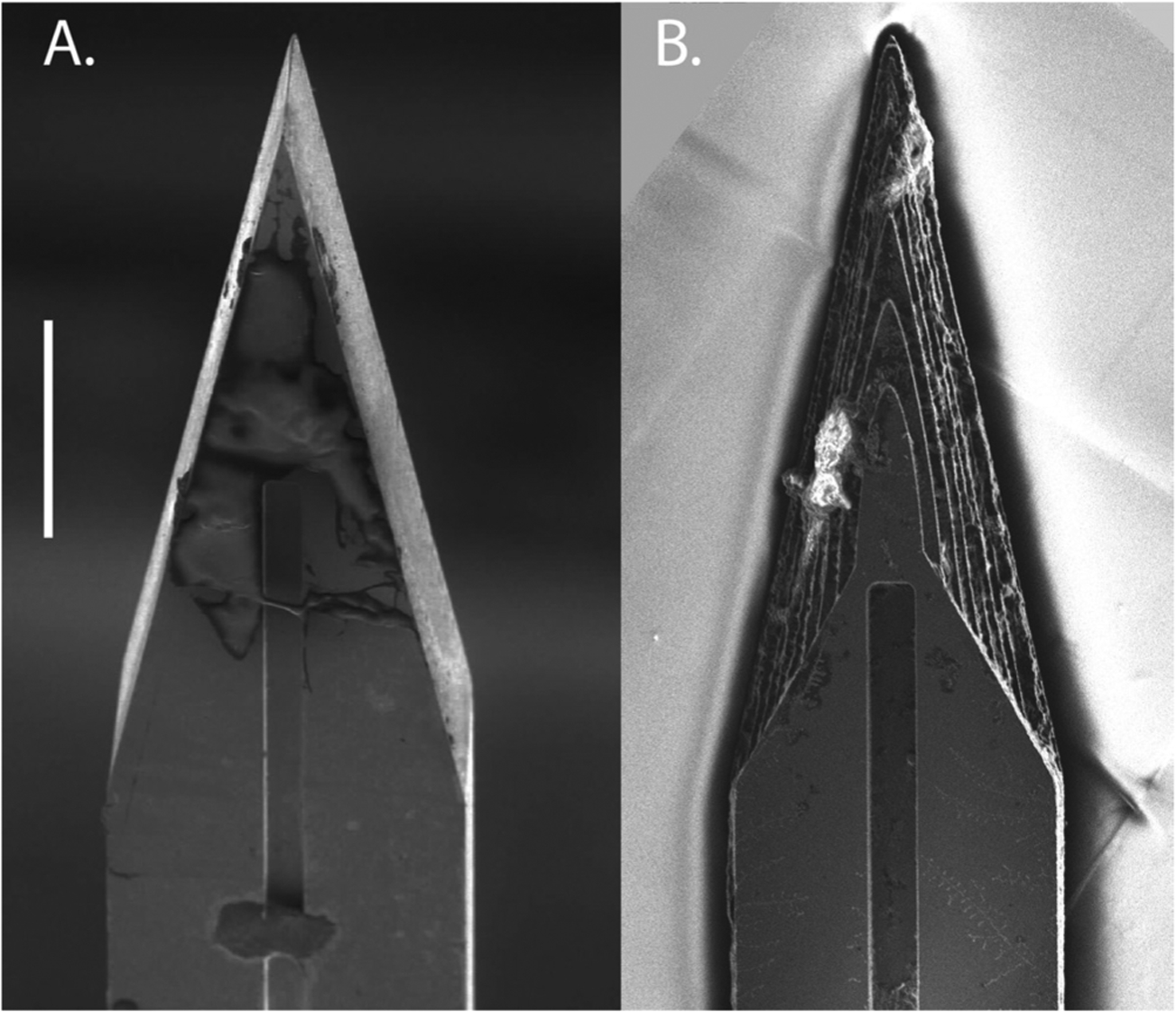
Top-down view by SEM for comparison of planar (A) and sharpened (B) shuttles (scale bar = 50 *μ*m). Shuttles are oriented with tips toward the top of the figure and wicking channel for PEG at midline. Residue in (B) from previous insertion through neural tissue.

**Figure 3.**
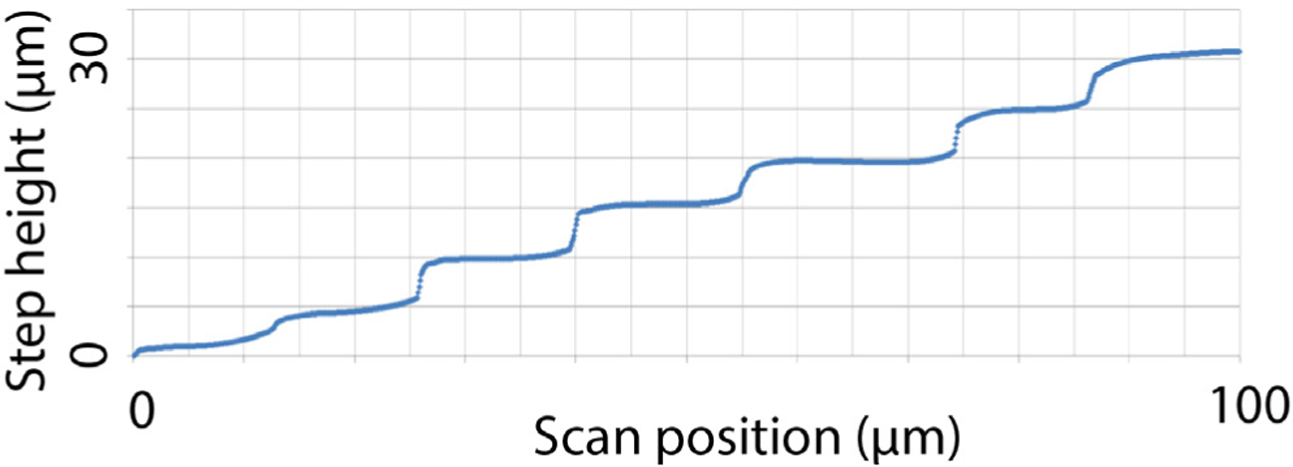
Profilometer scan of sharpened shuttle tip.

**Figure 4.**
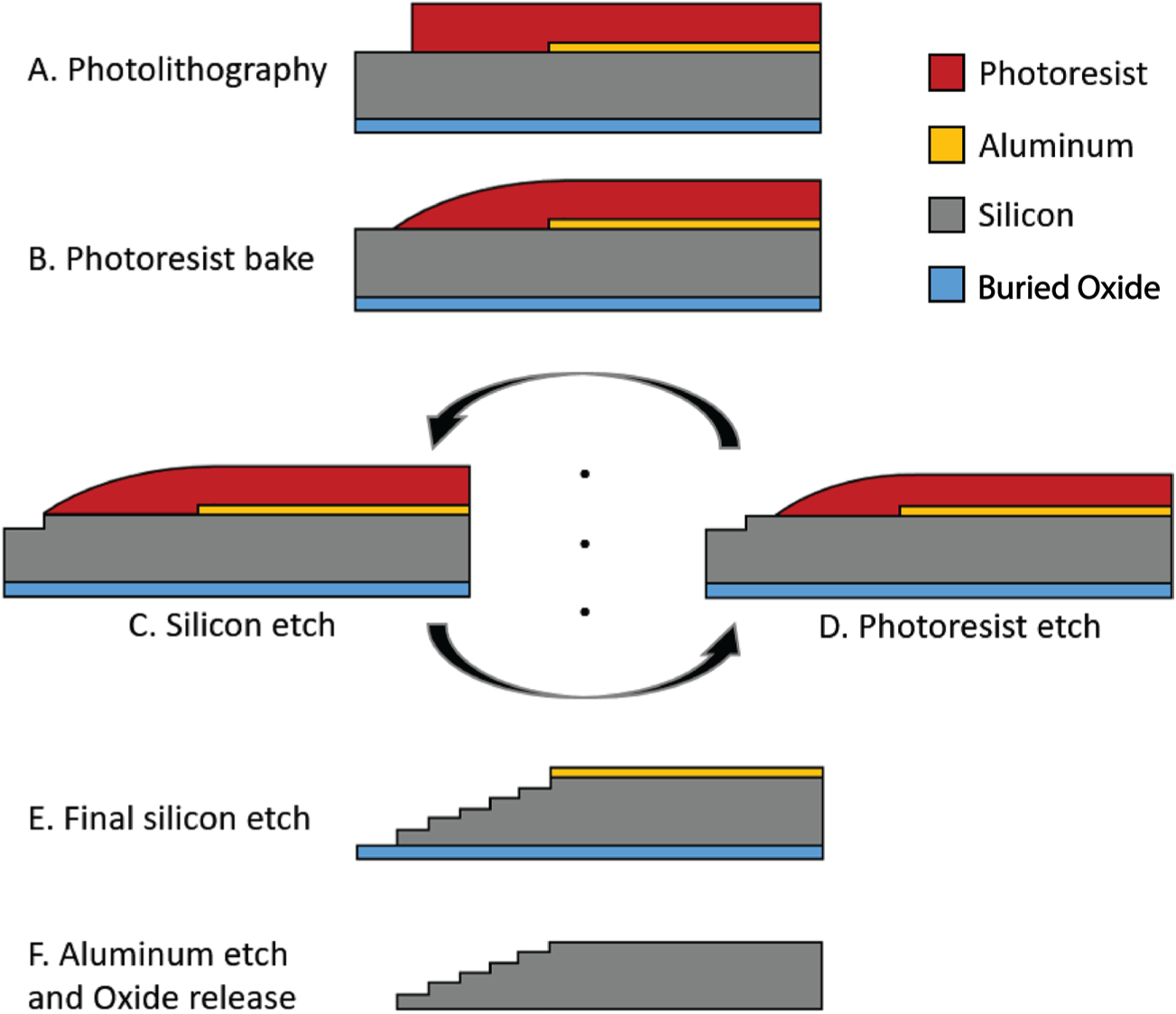
Process flow detail.

Planar and sharpened shuttles both had a 30° tip angle (figure 1), a width of 80 *μ*m, and a thickness of 30 *μ*m. The difference in their design was the gradual increase in thickness (the ‘sharpened’ profile) of the 3d-sharpened shuttle. Figure 2 shows scanning electron microscope (SEM) images of planar (figure 2(a)) and sharpened shuttles (figure 2(b)) mounted on double-sided copper at magnification 300 × with accelerating voltage 3 kV using an APREO S Low Vacuum EDX Benchtop SEM (figure 2(a)) or at magnification 650 × with a Hitachi S-800 SEM (figure 2(b)). Figure 3 is a profilometer scan, taken using a Veeco Dektak profilometer, in which the scan proceeds from the shuttle tip along the shank as it increases in thickness. For this representative shuttle, seven individual step heights, each approximately 5 *μ*m higher than the previous, are seen over the 100 *μ*m length of the tip. Both planar and sharpened insertion shuttles were fabricated on 4″ silicon-on-insulator (SOI) wafers with 30 *μ*m device layers (figure 4). First, 10 *μ*m deep ‘wicking’ channels (figures 1 and 2) were fabricated using standard photolithography and the bosch deep reactive ion etch (DRIE) process in an STS ICP DRIE tool (SPTS Technologies). Wicking channels were designed to hold polyethylene glycol (PEG, molecular weight 10 000 mn), which acted as a biodissolvable adhesive to mount polymer probes on insertion shuttles, as described in Felix *et al* (2012). Next, 300 nm of aluminum was sputtered (Semicore Equipment Inc.), patterned photolithographically, and wet etched to create a hard mask with the insertion shuttle geometry. Sharpened shuttle fabrication involved a third photolithography step to specify the sharpened tip profile overlaid on the aluminum hard mask, followed by a 2 minute reflow bake of the photoresist (AZ4620) at 180° Celsius (figures 4(a) and (b)). This resulted in a sloped profile in the patterned photoresist that was transferred to silicon using alternating timed silicon etch and photoresist etch steps (figures 4(c), (d) and (e)) in the same STS plasma etcher. The height of each ‘stair’ shown in figure 3 is determined by a timed silicon DRIE step, which alternates SF_6_ for etching and C_4_F_8_ for passivation. The length of each stair is determined by both a timed photoresist etching step using O_2_ and the photoresist reflow step (figure 4(b)). Uneven height and length of the stairs can arise from either imperfect photoresist reflow or imperfect timing of etching steps. Dimensions of the stairs can be adjusted to etch arbitrary 3D profiles, in contrast with anisotropic wet etching methods for which angles are restricted by silicon crystal structure. Planar shuttles under-went DRIE without the third photolithography step, resulting in a tip profile of uniform thickness. Lastly, the aluminum hard mask was removed (Piranha etch) and individual shuttles were released from the SOI wafer using hydrofluoric acid.

### 1.2. Polymer probe microfabrication

**Figure 5.**
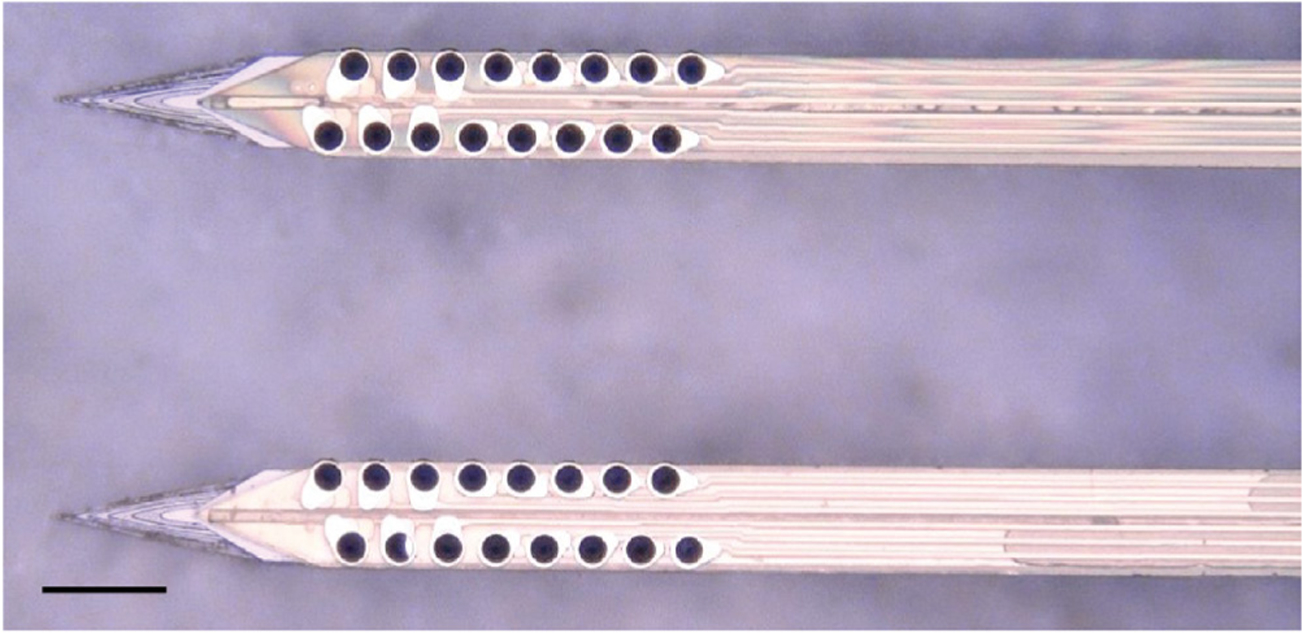
Top-down view by light microscope of a single, two-shank, 32-channel probe mounted on a sharpened, two-shank silicon shuttle. The shuttle tip extends approximately 100 *μ*m beyond the tip of the probe, which appears yellow-gold atop the shuttle and has, for each shank, two offset rows of eight PEDOT-PSS plated platinum contacts that appear dark. The distance between the shanks is 250 *μ*m. Scale bar = 100 *μ*m.

Polymer devices used in chronic recordings were made of polyimide with platinum electrodes electroplated with PEDOT:PSS. Each device had two shanks separated by 250 *μ*m, each 80 *μ*m wide and 6 or 8 mm long. As shown in figure 5, each shank had 16 contacts arranged in an offset dual-line configuration of 8 contacts each. Contacts were 20 *μ*m in diameter with 20 *μ*m contact edge-to-edge spacing and 6 *μ*m from electrode edge to shank edge. The image in figure 5 was taken using Keyence Model VH-Z250R at ~250×, with illuminated lighting on auto detect for white balance and brightness.

### 1.3. Surgical insertion in vivo

We used single-shank silicon shuttles (figure 1) for *in vivo* insertion force tests. Single-shank shuttles were created for shuttle-only tests by cutting off one of the shanks on a two-shank shuttle. All animal-involved protocols described in this manuscript have been approved by the Institutional Animal Care and Use Committee at UCSF. Studies were performed in male Long Evans rats. For the comparison of force insertion measurements between planar and sharpened shuttles *in vivo*, two animals were used, ages 12 and 16 months. For sharpened shuttle-guided insertion of probes for chronic neural recordings, one animal, age 10 months, was used.

In all cases, animals were anesthetized with inhaled isoflurane plus a mixture of ketamine, xylazine, and atropine and were confirmed unresponsive to a foot pinch. The head was shaved and the animal transferred to the sterile surgery environment and head-fixed in a stereotactic frame. Body temperature was maintained throughout surgery by an isothermal pad beneath the animal. Following sterilization of the incision site and application of lidocaine as a local anaesthetic, a minimal incision was made through all skin layers at the top of the skull. The skin flaps were retracted and the tissues detached from the bone, preserving the attachment of the temporalis muscle to the temporal ridge. For the chronic recording animal, a dental drill was used to make 12 sub-penetrating skull holes along the temporal ridge. Into each of these holes, 0–80 titanium set screws (United Titanium, OH) were partially inserted, serving to anchor the implant to the skull. These were drilled with a rotating dental drill with carbide bur (SS White carbide bur, FG 2) at 6000 rpm, at an intermittent rate, with the drill removed completely from contact with the bone between bouts of drilling. Coordinates for target insertion sites were located using bregma and stereotactic coordinates. One craniotomy of approximately 2–3 mm diameter was made to expose the meninges over each of NAc (1.5 AP, ± 1.3 ML, mm) and OFC (+3.6 AP, ±3.4 ML, mm) bilaterally (for chronic recordings), or each of hippocampus (−3.6 AP, ±2.5 ML, mm) and OFC bilaterally (for force insertion measurements). The craniotomy technique was the same as for skull screw holes with the exception that the final ~500 *μ*m of bone was removed using a smaller carbide bur (SS White carbide bur, FG ¼). The dura mater was maintained fully intact. Following *in vivo* data collection, animals were sacrificed using an overdose of pentobarbital sodium and phenytoin sodium (Euthasol, Virbac AH, Inc.).

### 1.4. Insertion force measurements

Insertion force tests of shuttles without attached devices were performed over hippocampus and OFC bilaterally in each of two animals. We performed 16 insertions in Animal 1 (four sharpened and four planar to hippocampus; four sharpened and four planar to OFC) and 11 insertions in Animal 2 (four sharpened and two planar to hippocampus; two sharpened and three planar to OFC). Each test insertion was performed with a new, single-shank shuttle over a new area of dura.

**Figure 6.**
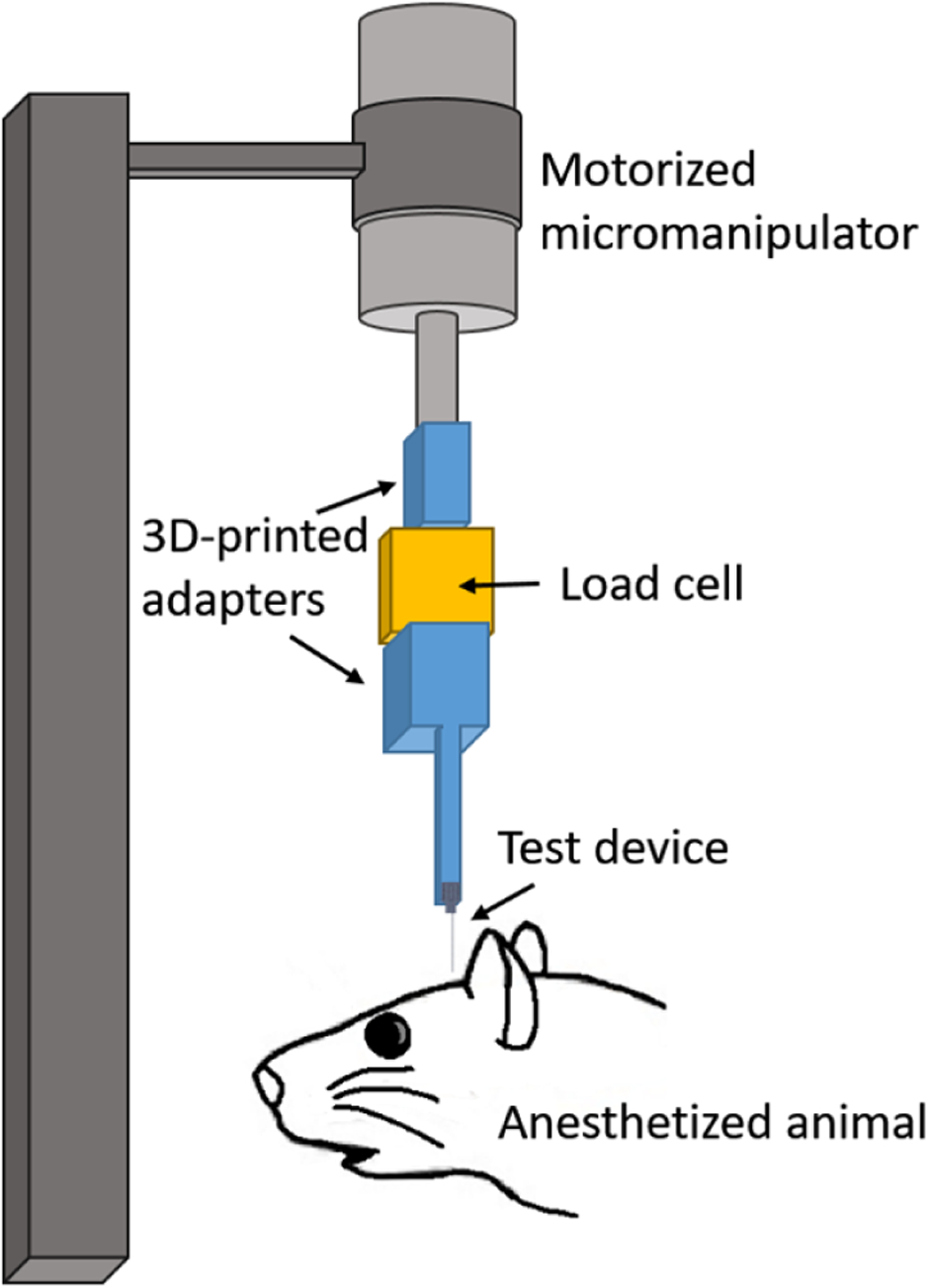
Test insertion apparatus for *in vivo* insertion force measurements. The test device was a single shank, 6 mm long.

**Figure 7.**
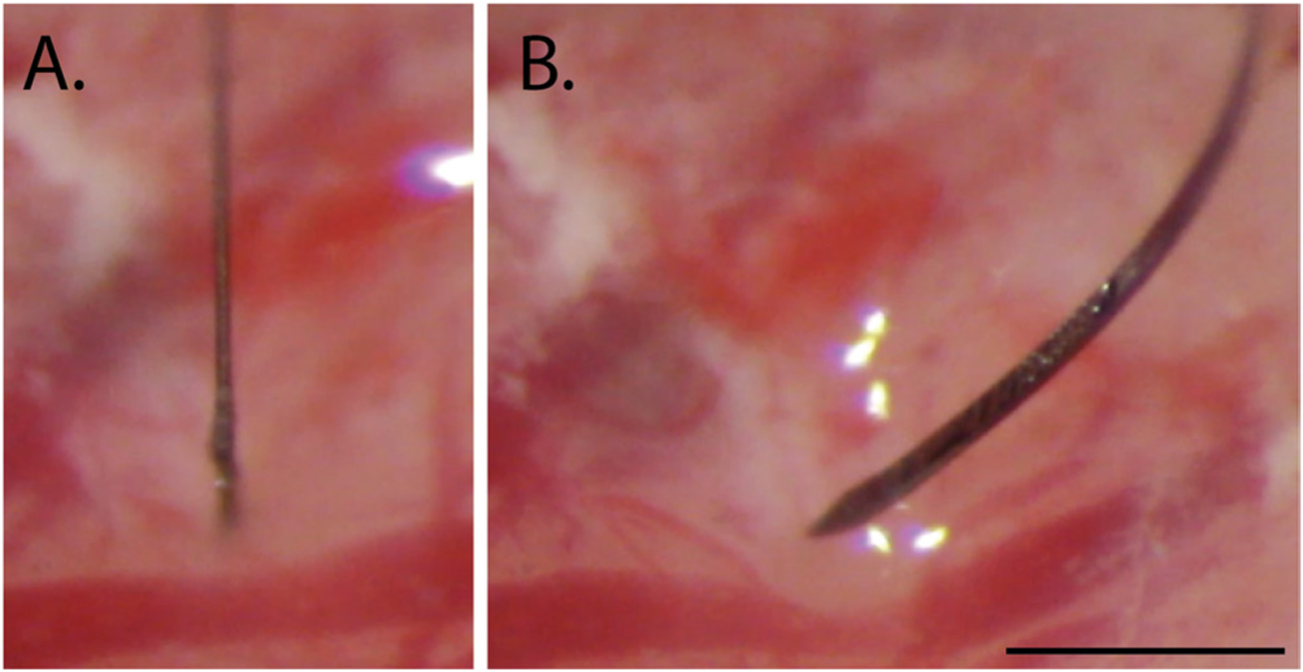
Example of a successful (A) and failed (B) insertion attempt for sharpened silicon shuttles, 6 mm. Scale bar = 1 mm.

In both experiments, insertion force measurements were performed with a precision load cell (FUTEK FSH02534) and voltage was read out through the digitizer (FUTEK FSH03633) to a computer at 100 samples s^−1^. The largest source of error in our force measurements is in the readout of the digitizer, which is load-dependent and equivalent to ±0.067 mN for our measured loads. Rigid adapters between the motor, load cell, and insertion shuttle were 3D-printed with hard plastic from custom designs (PolyJetHD Blue, Stratasys Ltd.; figure 6). Insertions were conducted at a constant velocity of 50 *μ*m s^−1^ using a micropositioner (Kopf model #2662) and insertion force was measured as the shuttle contacted dura and either inserted or failed (figure 7). To match our typical surgical conditions, we tested serial insertions through dura in the same craniotomy, with at least 300 *μ*m separation between insertion sites. Each insertion was performed with a shuttle through intact dura. The dura and brain were hydrated for the duration of the surgeries using hand irrigation with saline.

Shuttle insertions were classified as successes if they inserted through dura without signs of buckling. Consequently, insertions were classified as failures if they visibly buckled, regardless of whether they eventually penetrated dura.

Maximum insertion force was measured at the time of penetration, which was taken as the first absolute maximum of the force curve that preceded the first approximately infinite-slope curve characteristic of membrane penetration (Sridharan *et al* 2013, Obaid *et al* 2018). In a few cases, a slightly higher force was measured after initial penetration. In these cases we did not use the later timepoint to determine the maximum insertion force because, by then, the device had already begun to penetrate tissue and the total force measured thus included the frictional force as well.

Brain deflection distances were calculated by multiplying the insertion speed (50 *μ*m s^−1^) by the time between dural contact and penetration. We estimated the time of dural contact by first calculating the mean and standard deviation of the (150-sample minimum) baseline period, during which the shuttle was still above tissue and contacting only air; we then determined the point at which a five-sample sliding average of the measured force reached at least one standard deviation above the baseline mean. The time of penetration was chosen to be the first large peak of the force trace as described above. The load cell itself compresses as a function of force applied, and the amount of compression was measured under a microscope and subtracted from the brain deflection values calculated from force measurement data as described above. This resulted in a correction of 5–12 *μ*m, which is within the variance of deflection values between insertions.

### 1.5. Imaging

Shuttles were imaged using Keyence Model VH-Z250R at ~1000×, with illuminated lighting on auto detect for white balance and brightness. Eight planar and eight sharpened shuttles were each imaged both before and after *in vivo* insertions through dura to verify that no shuttle breakage occurred during insertion or retraction.

### 1.6. Chronic recordings

All procedures were in accordance with guidelines from the University of California San Francisco Institutional Animal Care and Use Committee and US National Institutes of Health. For the tests of shuttles used with chronically implanted probes, the probe and shuttle were adhered to each other by PEG on the side opposite the recording contacts, as previously described for planar silicon shuttles. The meninges were hydrated between craniotomy completion and the time of insertion using hand irrigation with a saline drip. When all craniotomies were complete, a custom designed, 3D-printed plastic base piece was secured to the skull using dental acrylic attached to the 128-channel headstage (SpikeGadgets, LLC). These insertions were performed manually, by lowering with a stereotax at approximately 50 *μ*m s^−1^ until the probe penetrated the dura mater. Once in the brain, the device was lowered using a micromanipulator (MO-10, Narshige) at 10 *μ*m s^−1^ until it was 1 mm above target depth, then at 5 *μ*m s^−1^ until it was 500 *μ*m above target depth, and at 3 *μ*m s^−1^ until it reached target depth.

The four implant targets were: OFC bilaterally, at the same coordinates for the insertion force measurements, at depth −4 mm from brain surface; and NAc at depth −7 mm from brain surface. Following insertion, probes were secured to the base piece and the electronics connected. Once the probe reached target depth, polyimide ‘wings’ glued to the probe perpendicular to its length were adhered to the base piece with acrylic, securing the probe at the target location. The shuttle was then detached from the probe by filling the base piece with saline, which dissolved the PEG at the probe-shuttle interface, thus allowing retraction of the shuttle without disrupting the probe. After all the probes were inserted, the base piece was drained of saline and filled with silicon elastomer (Dow Corning 3–4680) to prevent dural regrowth and scarring, brain swelling, and mechanical perturbation of the probes (Jackson and Muthuswamy 2008). Kwik-sil (World Precision Instruments, LLC), dental acrylic, and a plastic case made of moldable plastic (ThermoMorph) was used to encase the devices. Post-surgically, meloxicam (Eloxiject, Henry Schein) and buprenorphine (Baytril, Reckitt Benckiser Healthcare) were given for pain, and a single dose of enrofloxacin (Baytril, Bayer Corporation) was given to prevent infection. Neural recordings were conducted in an approximately 1 square foot sleep box constructed of anti-static plastic and located in a recording room. Data were collected for 30 minutes or longer per day using the SpikeGadgets recording system (Trodes version 1.7.4), as previously described (Chung *et al* 2019). The recording session analysed here was 1 hour long. Typically, the animal was asleep or quietly immobile during the recording period. The animal also ran a spatial navigation behaviour in epochs distinct from the sleep epoch analysed here. Recording quality was analysed at 95 d, as we have found previously that cell count using these polymer probes stabilizes by this time (Chung *et al* 2019).

### 1.7. Neural data analysis

Data pre-processing was performed using custom Python and Matlab scripts. Common-average referencing was applied across the sixteen channels of each shank. Spike sorting was performed using the MountainSort software package (Chung *et al* 2017, 2019), version 4.0. An initial round of automated sorting was performed with the following sorting parameters: detect sign = −1, detect threshold = 3, clip size = 100, adjacency radius = 200 *μ*m. The raw data were filtered between 600 and 6000 Hz. The detect interval was set to ten samples and the first ten principal components were used. For units identified using these parameters, only those with cluster quality metrics above the following thresholds were included as single units: firing rate threshold = 0.01 Hz, isolation threshold = 0.96, noise overlap threshold = 0.03, peak signal-to-noise ratio threshold = 1.5. The rest were marked as multi-unit activity. All identified units, including those marked as multi-unit activity, were manually inspected and curated: in MountainView software, clusters that did not appear to be single units based on refractory period violations (i.e. frequent spiking within 2 ms of the last spike) were rejected; multiple clusters that were identified as corresponding to the same unit based on a combination of firing rates, waveforms, peak channels, and temporal cross-correlograms were merged, and all cluster metrics listed above were re-calculated. Clusters that passed the curation metric thresholds were accepted.

## 2. Results

### 2.1. Planar and sharpened shuttle insertion forces

**Figure 8.**
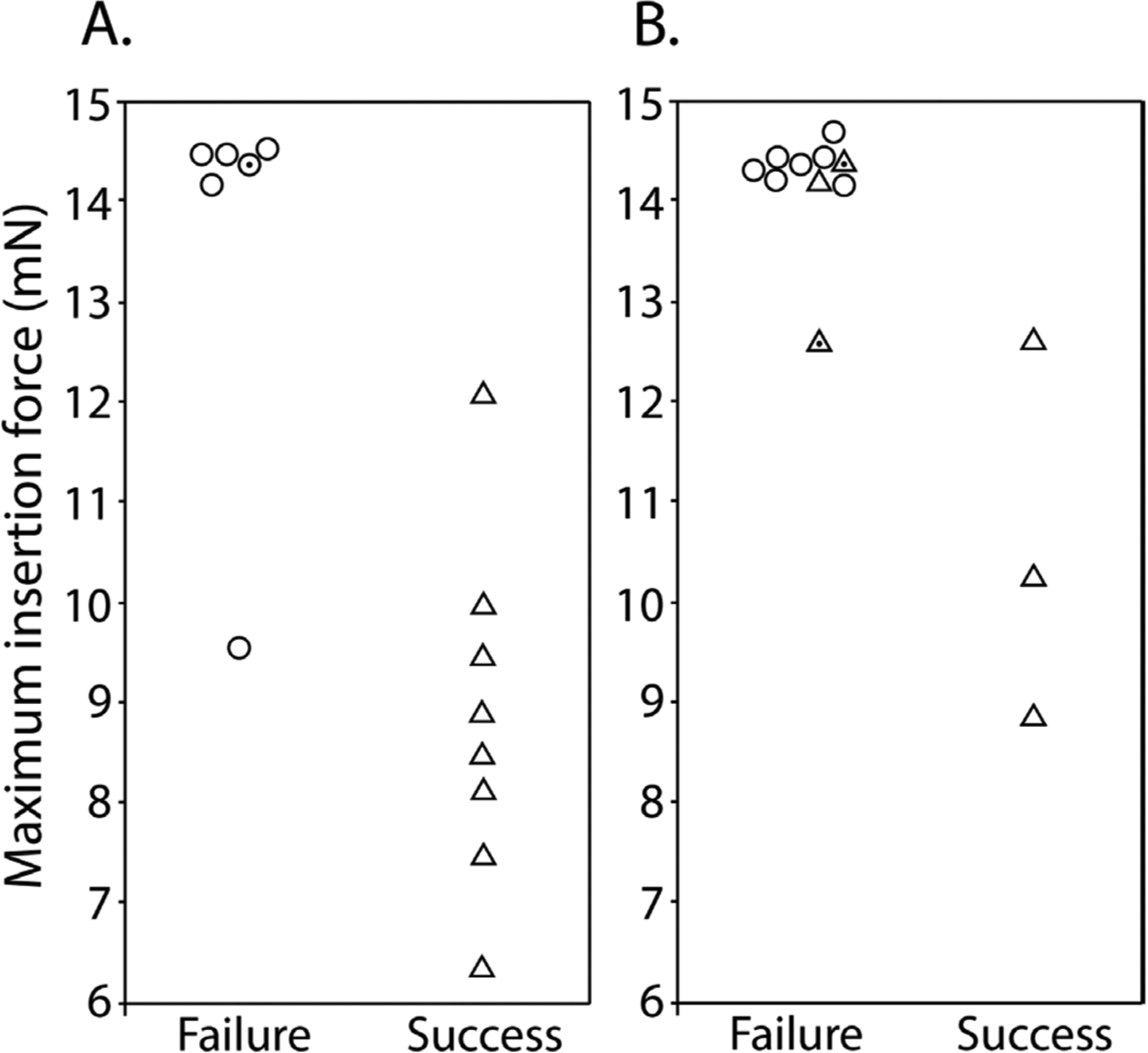
Maximum insertion force for sharpened (triangles) and planar (circles) shuttles inserted to (A) hippocampus (B) OFC that either failed (left) or succeeded (right). *Y*-axis = maximum insertion force in mN. Random jitter applied along *x*-axis for visualization of failed insertions. Points with dot centers indicate shuttles that penetrated dura but only after buckling to an extent that would likely have caused separation from an attached polymer probe.

We first tested single-shank shuttles with either a flat profile (planar; *N* = 6) or a sharpened profile (sharpened; *N* = 8) but otherwise identical dimensions (6 mm × 30 *μ*m × 80 *μ*m, figures 1 and 2) in transdural insertion to the hippocampus (figure 8(a)). Sharpened shuttles penetrated dura (8/8 shuttles, 100%) but planar shuttles did not (0/6 shuttles, 0%). Figure 8(a) shows the maximum insertion force for each insertion test over hippocampus. One shuttle characterized as *failed* did penetrate dura, but only after buckling to an extent that would likely have caused separation from an attached polymer probe due to the slight difference in curvature of the probe relative to the shuttle during buckling. For successfully inserted shuttles, the average insertion force was 8.9 ± 1.6 mN (*N* = 8, ± s.d.). In contrast, failed penetrations resulted in buckling and had an average buckling force of 13.6 ± 1.8 mN (*N* = 6, ± s.e.m.; figure 8(a), left).

**Figure 9.**
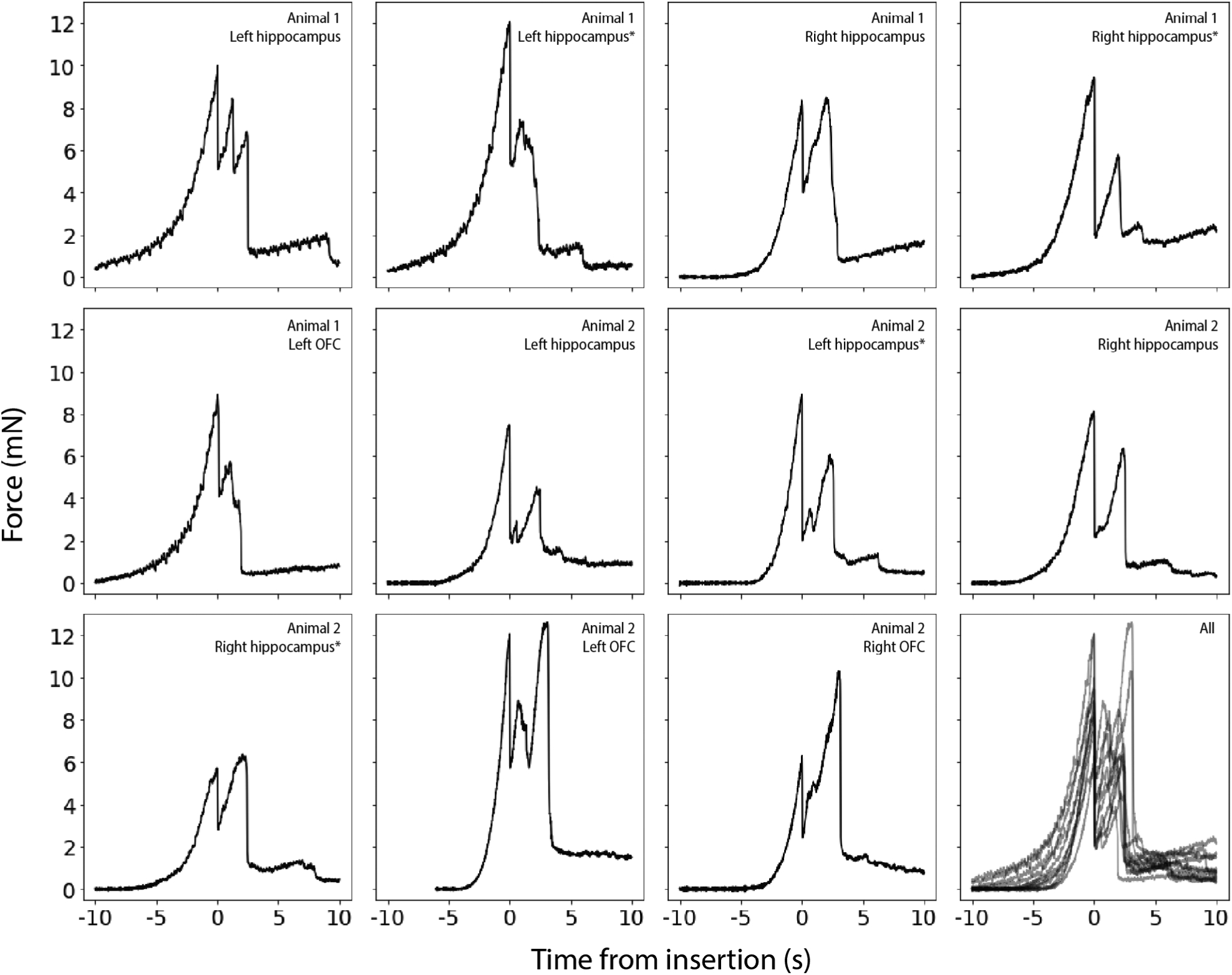
Raw force traces for all sharpened shuttles inserted through dura without buckling. *X*-axes in seconds, with traces aligned to 0 as the timepoint of maximum force, *y* -axes in mN. The animal and area of insertion are labelled in the upper right of each panel; plot at lower right shows all insertion force measurements depicted here overlaid. If the insertion was through a different area of a craniotomy where a section of dura at least 300 *μ*m away was already used for a test insertion, the insertion site is marked with an asterisk.

The insertion force traces for shuttles that successfully penetrated the dura showed increasing force, followed by a characteristic series of rapid decrements between local maxima, in a sawtooth pattern (figure 9). In a few cases, a slightly higher force was measured following initial penetration (figure 9, panels 3, 9, 10, 11 from top left to bottom right), likely due to increased friction force after the device had begun to penetrate tissue. In these cases we took the first local maximum as the maximum insertion force.

Our hippocampal target was located approximately halfway between bregma and lambda, and halfway between the midline and the temporal ridge. As a test of the effectiveness of our sharpened shuttles through thicker dura, we next performed the same tests over the OFC, where the dura is thicker and more difficult to penetrate. Tests were performed for OFC as for hippocampus (figure 8(b)). Of the sharpened profile shuttles tested, 3/6 shuttles (50%) penetrated dura, while 0/7 planar shuttles (0%) did. The worse performance over OFC relative to hippocampus is expected given the tougher dura. Figure 8(b) shows the maximum insertion force for each insertion test over OFC. Two sharpened shuttle insertions characterized as *failed*, one over left OFC and one over right OFC, successfully penetrated dura but did so only after buckling to an extent that would likely have caused separation from an attached device.

For successfully inserted shuttles over OFC, the average insertion force was 10.6 ± 1.5 mN (*N* = 3, ± s.e.m.; figure 8(b), right; insertion profiles also included in figure 9), which is not significantly different than for hippocampus (Welch’s *t*-test: *p* = 0.24). In contrast, failed penetrations included both sharpened and unsharpened shuttles and had an average buckling force of 14.2 ± 0.6 mN (*N* = 10, ± s.e.m.; figure 8(b), left).

### 2.2. Brain compression on transdural shuttle insertion

We calculated the degree of brain deflection for sharpened shuttles successfully inserted through dura without buckling (i.e. those insertions included in figure 9). Deflection distances were calculated using the number of samples between dural contact and penetration (refer to Methods for details). On average, the calculated compression for sharpened shuttle insertions through dura was 389.9 ± 159.6 *μ*m (*N* = 8, ± s.e.m.) for hippocampus and 302.5 ± 158.5 *μ*m (*N* = 3, ± s.e.m.) for OFC.

### 2.3. Imaging before and after insertion

**Figure 10.**
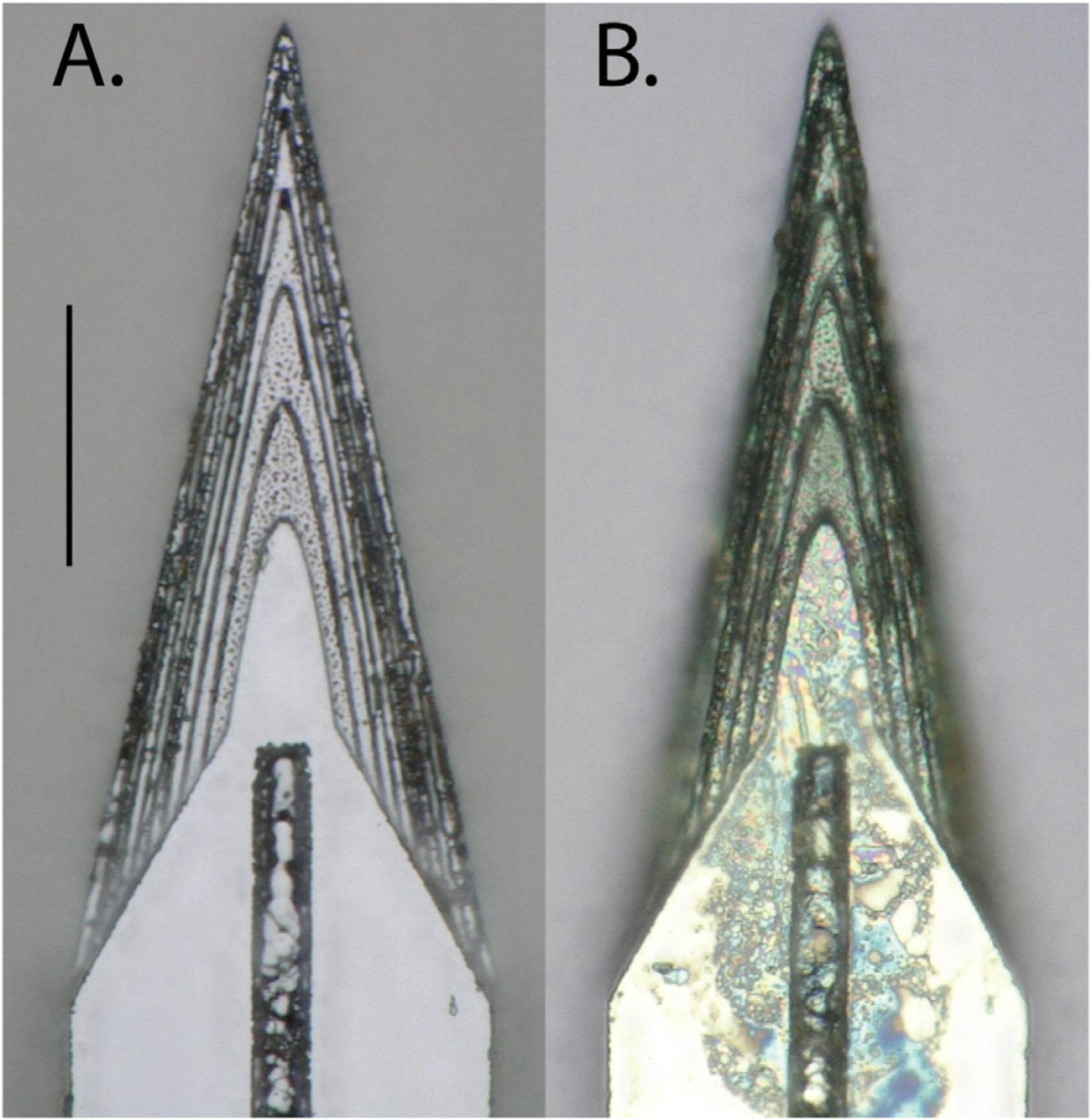
Top-down view of the same sharpened shuttle. (A) Before insertion. (B) After insertion through dura. Scale bar = 50 *μ*m.

We imaged eight sharpened and eight planar shuttles before and after *in vivo* insertion. We observed no breakage or other discernible damage to any of the shuttle tips, indicating that sharpened shuttles, despite having a tip diameter of less than 3 *μ*m in width and thickness, maintain their structural integrity and do not break off in the brain (figure 10).

### 2.4. Neural recordings using sharpened shuttles

Device-attached shuttles entered the brain with minimal compression of the dura and brain, with no observable difference relative to shuttle-only insertions. This is expected, given that devices were intentionally attached to shuttles such that their tips were shifted approximately 100 *μ*m back along the shuttle shanks (figure 5). This enabled the bare sharpened shuttle tip, without the device, to form the penetrating point. We observed that after the dura was punctured, tension in the membrane slightly expanded the opening to allow the probe, which itself is 14 *μ*m thick, to enter the brain. After dural penetration using a stereotax, the device was inserted to its final depth using a micromanipulator.

**Figure 11.**
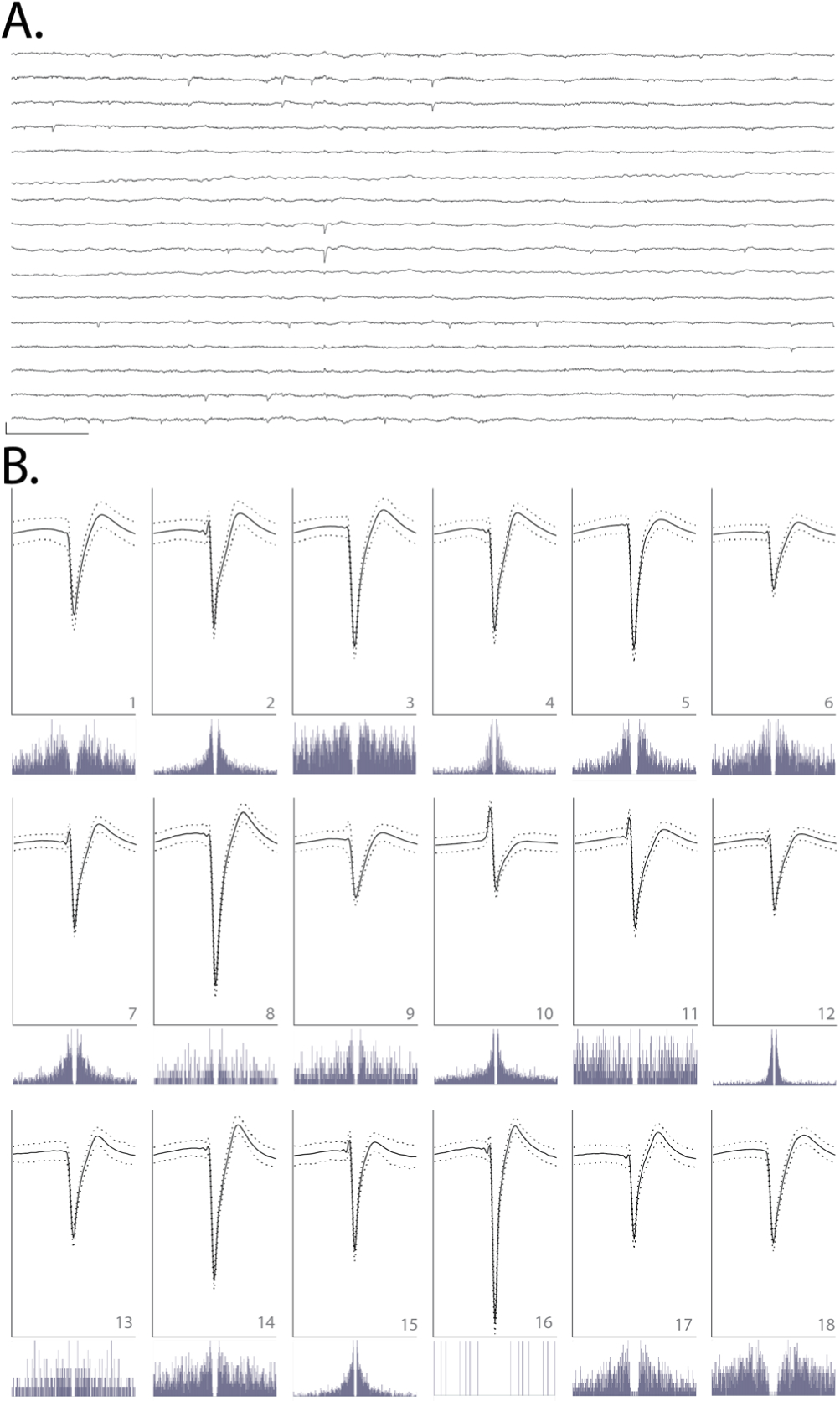
(A) Raw, 150 ms local field potential recorded on each of the 16 channels of one shank targeted to OFC, 95 d after implant. Channels 1–16 are ordered from top to bottom. Channels 1–8 correspond to electrodes on the left side of the probe shank, from top to bottom; channels 9–16 correspond to electrodes on the right side of the device, from bottom to top (i.e. channels 1 and 16 are at similar depth). Scale bar, bottom left = 1 mV vertical, 15 ms horizontal. (B) Each of 18 units detected on the 16-channel shank shown in (A). Odd rows: average waveforms (solid line) ± one standard deviation (dashed line) for units 1–18, numbered in bottom right of each panel. For each waveform, vertical scale bar = 2.5 millivolts, horizontal scale bar = 100 samples (at 30 kHz), or approximately 3.33 ms. Even rows: spike auto-correlograms for the unit shown above, spanning 100 ms, in 0.5 ms bins.

We observed that single units were detected on all six implanted shanks (16 channels each) for at least 90 d. We selected a single epoch on day 95 post-implant to perform spike sorting on one of the shanks targeted to the left OFC. This shank was selected for its electrode quality prior to insertion (no shorts or dead channels) and relatively low noise levels (figure 11(a)). We identified 18 single units on this 16-channel shank, for an average of approximately one unit per electrode (figure 11(b)). This is similar to what we have previously observed for polymer arrays inserted using planar shuttles (Chung *et al* 2019). Single units that passed curation standards were evenly distributed over the recording channels, with no apparent systematic difference in quality, from the top to bottom of the shank.

## Discussion

Previous work has demonstrated the benefits of sharpened device tips in reducing insertion force (Sharp *et al* 2009, Fekete *et al* 2015, Obaid *et al* 2018) and penetrating dura (Hosseini *et al* 2007, Sharp *et al* 2009, Fekete *et al* 2015). Here, we sharpen not only in two dimensions but three, resulting in a more gradually increasing cross-section (Fekete *et al* 2015, Chen *et al* 2017). This is, to our knowledge, the first demonstration of a dural-penetrating, polymer device insertion method to yield chronic single unit recordings. It also allowed us to directly compare shuttles made of the same material (silicon) that were sharp in three versus two dimensions.

We have shown that sharpened silicon shuttles result in low insertion forces and brain compression. The expected buckling force, also known as the critical load, of our silicon shuttle can be calculated with Euler’s critical load equation:

(1) $$F_{\text{crit}}\;=\;\frac{\pi^{2}EIn}{L^{2}}$$

where *E* is the Young’s modulus of silicon, *I* is the moment of inertia for the cross-sectional area of the shuttle, *n* is a factor accounting for end conditions, and *L* is the length of the shuttle. The moment of inertia *I* of a rectangular cross-section is *ab*^3^*/*12 where *a* and *b* are the longer and shorter side lengths, respectively. We take 169 GPa as the Young’s modulus of our silicon shuttles (Hopcroft *et al* 2010), which are fabricated in the 〈1 1 0〉 crystal directions. For translation fixed, rotation fixed (probe side) and translation fixed, rotation free (brain side) end conditions, *n* = 2 and the expected critical load is

(2) $$F_{\text{crit}}\;=\;\frac{\pi^{2}\left(169\;\text{GPa}\right)\;\times \;\left(80\;\mu \text{m}\;\times \;\frac{{\left(30\;\mu \text{m}\right)}^{3}}{12}\right)\;\times \;2}{\left(6\;\text{mm}\right)}\;=\;16.7\;\text{mN.}$$

This is close to our measured result of 13.6 mN for hippocampus and 14.2 mN for OFC. Due to the deformable nature of the brain, the boundary condition on the brain side is not completely translation fixed, with true *n* less than 2 and true *F*_*crit*_ expected to be slightly lower than calculated. We did not, however, visually observe the shuttle tip to translate across the dural surface during insertions.

Our maximum insertion force for transdural insertion of sharpened shuttles of aproximately 10 mN is lower than previous reports for planar devices (e.g. 41 mN with silicon probes (Hosseini *et al* 2007)) and similar to the 11 ± 2 mN reported previously in a proof-of-concept study in which a less flexible, silicon-specific etch technique was used to insert but not record from silicon probes (Fekete *et al* 2015). Table 1 summarizes previously published insertion force measurements made through rat dura. For comparison, previously reported peak insertion forces for rat pia-only insertions are approximately 5 mN (Paralikar and Clement 2008, Fekete *et al* 2015). In addition to those devices included in the table, other silicon arrays have been inserted transdurally in cat (Rousche and Normann 1992) and rat (Escamilla-Mackert *et al* 2009) without measurement of insertion force. For comparison, commercially available silicon Neuronexus probes range from 15–50 *μ*m thick and the state-of-the-art silicon Neuropixels probe is 70 × 20 *μ*m (Jun *et al* 2017). These dimensions are comparable to the smallest devices in the table. The design and fabrication technique presented here could be used for these silicon electrode arrays to enable dural penetration.

Our insertion force traces each show multiple peaks separated by approximately 100 *μ*m in depth, which in some cases (figure 9, traces 3, 9, and 11 from the upper left) include a peak that is higher than the first. Additional force peaks over the course of insertion could be due to penetration of the pia and arachnoid mater, which lie between the dura mater and brain parenchyma. Such a sawtooth or non-monotonic pattern has been observed previously by other experimenters performing device insertions into the brain (Sridharan *et al* 2013, Obaid *et al* 2018) though not in all cases (Sharp *et al* 2009, Fekete *et al* 2015, Na *et al* 2018), and is expected to depend on the shape of the device and its insertion speed. In cases where such a pattern has been observed, it has been suggested that myelinated axon bundles or other heterogeneities in the brain parenchyma itself, below the insertion site, could account for the insertion force profile (Obaid *et al* 2018). Another possibility is that this pattern would be observed even in absence of the underlying brain parenchyma. The dura is a dense irregular connective tissue, made of collagen and elastin, and it is conceivable that the first peak on the insertion force trace could correspond to the tip of the shuttle breaking superficial matrix fibers, with subsequent peaks corre sponding to further breakage of deeper fibers. Such a process has been observed for rat dura-only penetrations (Maikos *et al* 2008). Heterogeneities in the brain parenchyma and dura exist not only across depths at a given insertion site, but at different anteroposterior and mediolateral coordinates. This may explain the variation in force traces for insertions performed at different locations within the same craniotomy, which will present different vasculature, cellular composition, and over-lying dural matrix fibers.

Recently, an insertion method using a diamond shuttle tested rat transdural insertion at a variety of speeds, finding a minimum average compression of 488 *μ*m at insertion speed 10 *μ*m s^−1^ with 200 Hz vibration (Na *et al* 2018). At approximately 300–400 *μ*m of compression without piezovi-bration, our method is comparable. To our knowledge, these are the lowest brain compression values for a working device through dura. It is a reasonable expectation that for surgeries where performing a durotomy is preferable, these devices can be implanted into the brain through pia with even lower brain compression. This use could be particularly valuable for superficial cortical sites in which recording quality is easily compromised by compression of the brain (Rennaker *et al* 2005).

Force and tissue compression are important metrics to gauge neural tissue damage, but a single unit count weeks after implant is required to fully demonstrate a neural recording technology for chronic use. Recently, promising strategies for insertion of flexible polymer devices through rat dura have been demonstrated (Shoffstall *et al* 2018, Zhao *et al* 2019), but such methods have not yet reported chronic, single-unit recordings in freely behaving animals. Here, we evaluated our single unit count at 95 d. At that timepoint we were able to record high quality single units on 11 of 16 (69%) channels, with a total of 18 sorted units on 16 channels, or an average of 1 high-quality unit per electrode. In comparison, the other recent technology that reports a single unit count is the dural penetrating diamond shuttle for delivery of a flexible array, which yielded acute neural recordings of 20 units over 60 channels, or an average of 0.33 units per electrode (Na *et al* 2018). Another alternative to manual durotomy is laser microablation, which has been used preceding robotic insertion of thin-film polymer probes. The longest recordings reported using this method were taken at two months post-implant, with approximately 40 percent of channels recording single-unit action potentials (Hanson *et al* 2019).

Further reduction in brain compression may be achieved by fabrication of even sharper profile tips (Obaid *et al* 2018), which our fabrication method can achieve with minimal adjustment. Another way to reduce brain compression could be to change insertion speed. A range of speeds, including meters-per-second scale pneumatic insertion (Black *et al* 2018), have yielded dural penetration (Fekete *et al* 2015) and neural recordings (Rousche and Normann 1992, Hosseini *et al* 2007, Zhao *et al* 2019), with the optimal speed dependent on tip shape and features in the target tissue, such as vessels (Bjornsson *et al* 2006). A recent report indicates that slower insertion of silicon probes into brain following durotomy, over the range 2 *μ*m s^−1^ to 1 mm s^−1^, improves cell yields in acute recordings (Fiath *et al* 2019). Retraction speed, too, has been varied; for example, the ballistic retraction method used in combination with needle-and-thread insertion to prevent displacement of electrodes from target depth has, as previously discussed, yielded chronic neural recordings (Hanson *et al* 2019). In addition to determining the optimal insertion and retraction speeds, incorporating fast axioaxial vibration with relatively slow insertion speed could also aid in insertion with minimal brain compression (Na *et al* 2018). Additionally, application of collagenase to the dura could make it more easily penetrable (Paralikar and Clement 2008), though this process is slow and titration to determine an appropriate concentration presents a challenge, as the dural membrane thickness varies with target location and age (Fekete *et al* 2015).

Another complementary insertion strategy uses an insertion guide, as recently demonstrated for insertion of SMP devices through rat dura (Shoffstall *et al* 2018). Inspired by the labium that guides the proboscis of the female mosquito, a guide at the insertion site increases the critical buckling load of the inserted device itself. Such a strategy could be used in combination with the sharpened shuttles validated here.

This paper addresses successful dural insertion with single and double-shank devices, but the fabrication method described here could be applied to larger, multi-shank arrays for recording more neural data, over a more distributed brain volume. If the spacing between shanks is much larger than the dimpling radius, we would expect each shank to experience the same force as if it were implanted independently. However, if the spacing between shanks is small, the required insertion force for the array is expected to scale with the number of shanks, as has been shown for probes from one to ten shanks (Hosseini *et al* 2007). The mechanics are akin to those that protect the yogi lying on a bed of nails, across which the total force is distributed to prevent injury. One array design that could maintain the ability to penetrate dura would have shanks of varied lengths such that, at any given time, only a subset of shanks is in the process of penetrating dura. Our measurements of brain deflection can guide the design of these arrays to determine these varied shank lengths. Although we have focused on the delivery of flexible arrays, the design and fabrication technique presented here could be used for silicon electrode arrays themselves, either for dural penetration or to reduce brain compression in clinical and non-human primate implants.

## Conclusion

Recording more neurons in distributed circuits will likely lead to scientific insights that are unachievable with a smaller number of neurons (Buzsaki 2004, Stevenson and Kording 2011). Polymer probes are among the most promising methods for chronic, large-scale neural recordings, but their insertion through the tough protective membranes of the central nervous system is challenging and currently limits their broad use and effectiveness. Here, we have validated for chronic recordings the first dural-penetrating shuttle in combination with a modular polymer probe-based recording platform. This method shows limited brain compression and obviates the need for a durotomy in rats and other model organisms with similar dural tensile strength. Maintaining intact dura will reduce post-surgical edema, likely increasing accuracy in depth-targeting of the electrode arrays. This is critical for fixed, non-drivable arrays, particularly for targets with a small dorsoventral extent. The number and quality of single units we have recorded with this system is comparable to what we have previously recorded in the OFC using planar shuttles, inserted through pia only (Chung *et al* 2019). It is our hope that this method to more efficiently implant polymer devices for high-density, chronic neural recordings will enable experimentalists to address compelling open questions in neuroscience.

## Figures and Tables

**Table 1.** Rat trans-dural insertion forces measured in this paper and in previously published work.

	This paper	Hosseini *et al* (2007)	Fekete *et al* (2015)	Na *et al* (2018)
Material	silicon	silicon	silicon	UNCD
Dimensions (*μ*m)	30 × 80	100 × 120	100 × 500	11 × 65, + 27.5 × [16 ▹2] (tapered vertical support)
Shape	3D-sharpened tip < 3 *μ*m × 3 *μ*m	planar sharpened	chemically sharpened to a tip	planar sharpened
Insertion speed (*μ*m s^−1^)	50	—	50	10
Insertion force (mN, ± s.d.)	8.86 ± 1.73	41 ± 25.5	11 ± 3	6.67 ± 2.16
Insertion site	hippocampus	somatosensory cortex	various, posterior to bregma	motor cortex
Simultaneous single units : electrodes	18:16 (chronic)	—	—	20:60 (acute)
